# Recreational Exposure during Algal Bloom in Carrasco Beach, Uruguay: A Liver Failure Case Report

**DOI:** 10.3390/toxins9090267

**Published:** 2017-08-31

**Authors:** Flavia Vidal, Daniela Sedan, Daniel D’Agostino, María Lorena Cavalieri, Eduardo Mullen, María Macarena Parot Varela, Cintia Flores, Josep Caixach, Dario Andrinolo

**Affiliations:** 1 Italian Hospital of Buenos Aires, C1199ABB Buenos Aires, Argentina; flavia.vidal@hospitalitaliano.org.ar (F.V.); daniel.dagostino@hospitalitaliano.org.ar (D.D.); maria.cavalieri@hospitalitaliano.org.ar (M.L.C.); eduardo.mullen@hospitalitaliano.org.ar (E.M.); maria.parot@hospitalitaliano.org.ar (M.M.P.V.); 2 Area of Toxicology, Centre for Environmental Research, Exact Sciences Faculty, National University of La Plata, National Council of Scientific and Technical Research (CIMA-FCE-UNLP-CONICET), 1900 La Plata, Buenos Aires, Argentina; danielasedan@yahoo.com.ar; 3 Mass Spectrometry Laboratory/Organic Contaminants, Institute of Environmental Assessment and Water Research (IDAEA), CSIC, 08034 Barcelona, Spain; cintia.flores@idaea.csic.es (C.F.); josep.caixach@idaea.csic.es (J.C.)

**Keywords:** cyanobacteria, microcystins, recreational exposure, liver failure

## Abstract

In January 2015, a 20-month-old child and her family took part in recreational activities at Carrasco and Malvín beaches (Montevideo, Uruguay). An intense harmful algae bloom (HAB) was developing along the coast at that time. A few hours after the last recreational exposure episode, the family suffered gastrointestinal symptoms which were self-limited except in the child’s case, who was admitted to hospital in Uruguay with diarrhea, vomiting, fatigue, and jaundice. The patient had increased serum levels of liver enzymes and bilirubin and five days later presented acute liver failure. She was referred to the Italian Hospital in Buenos Aires, being admitted with grade II–III encephalopathy and hepatomegaly and requiring mechanical respiratory assistance. Serology tests for hepatitis A, B, and C, Epstein-Barr virus, and cytomegalovirus were negative. Laboratory features showed anemia, coagulopathy, and increased serum levels of ammonium, alanine aminotransferase (ALT), aspartate aminotransferase (AST), and bilirubin. Autoimmune Hepatitis Type-II (AH-II) was the initial diagnosis based on a liver kidney microsomal type 1 antibodies (LKM-1) positive result, and twenty days later a liver transplant was performed. The liver histopathology had indicated hemorrhagic necrosis in zone 3, and cholestasis and nodular regeneration, which were not characteristic of AH-II. LC/ESI-HRMS (liquid chromatography electrospray ionization high-resolution mass spectrometry) analysis of MCs in the explanted liver revealed the presence of Microsytin-LR (MC-LR) (2.4 ng·gr^−1^ tissue) and [D-Leu^1^]MC-LR (75.4 ng·gr^−1^ tissue), which constitute a toxicological nexus and indicate a preponderant role of microcystins in the development of fulminant hepatitis.

## 1. Introduction

Toxigenic cyanobacteria blooms favored by environmental conditions and eutrophication of water bodies worldwide occur periodically [1,2]. Cyanobacteria genera, such as *Microcystis*, *Anabaena*, *Oscillatoria*, and *Nostoc*, among others, are capable of producing a wide range of toxins, like microcystins, cylindrospermopsins, nodularin, and anatoxins [3]. The presence of cyanobacteria blooms in water bodies affects water quality owing to the production of cyanotoxins and odoriferous compounds. In the La Plata basin one of the most toxic and frequently-present cyanobacteria is *Microcystis* sp., often producing microcystin-LR (MC-LR) and [D-Leu^1^]MC-LR [4,5]. These toxins are considered among the most toxic hepatotoxins produced by cyanobacteria. 

Furthermore, the water bodies where blooms occur are commonly used for recreational purposes or as drinking water sources. People and animals can, therefore, be exposed to cyanobacteria and their toxins via several pathways. This worrying situation constitutes a major health and environmental hazard [3,6].

MCs have been associated with damage to human and animal health, causing different characteristic patterns of damage that depend on the route, intensity, and time of exposure. Reported diseases caused by cyanobacterial blooms and/or MCs vary in severity, ranging from death due to liver failure (Caruaru syndrome) [7,8] and gastrointestinal syndromes [9,10,11,12,13] to skin damage, respiratory problems [14,15], and liver cancer [16,17,18].

Recreational exposure to cyanobacterial blooms mainly involves oral, dermal, and inhalation pathways, depending on the type of activities undertaken in the water body. 

This type of exposure has been suspected in humans but not always confirmed, given the lack of detection of cyanotoxins in biological samples of exposed people and because cyanobacterial hepatotoxicosis is often underdiagnosed [19,20].

In this work we present a case of a family of three adults and a 20-month-old child who engaged in recreational activities, including bathing at the Carrasco and Malvín beaches in Montevideo, Uruguay, during the summer algal bloom in January 2015. 

A few hours after the last exposure episode, the family members developed gastrointestinal symptoms (diarrhea) which were rapidly self-limited in the three adults. The girl, however, continued to present gastrointestinal symptoms, such as diarrhea and vomiting, developing fatigue and jaundice, until finally, five days after exposure, she was admitted to the intensive care unit of a toxicology medical center (CIAT Uruguay). Laboratory tests showed increased serum levels of alanine aminotransferase (ALT: 1814 UI·L^−1^), aspartate aminotransferase (AST: 1946 UI·L^−1^), total bilirubin (8.15 mg·dL^−1^), direct bilirubin (4.8 mg·dL^−1^), and International Normalized Ratio (INR > 3). The patient was subsequently referred to the liver transplant center of the Italian Hospital in Buenos Aires, Argentina. Based on the epidemiological history of the patient, who had been bathing in the water with her family at the Carrasco and Malvín beaches (Montevideo, Uruguay) during a period of harmful algal bloom (HAB), the final diagnosis was acute liver failure related to cyanobacteria toxicity.

Our research group has performed histological studies and MCs determination in the explanted liver. We have also summarized the report on the environmental monitoring of Montevideo beach water quality undertaken by the authorities of Montevideo city between December 2014 and January 2015 [21]. 

The results indicate that the recreational exposure to MCs played a preponderant role in the development of acute hepatic failure suffered by the patient.

## 2. Results

### 2.1. Environmental Conditions of the Carrasco and Malvín Beaches in the Summer Season, 2014–2015

Montevideo (34°52′1′′ S, 56°10′0′′ W) is the capital city of Uruguay with a population of 1,319,108. This city has several beaches, including Carrasco, Malvín, and Pocitos, among others, in which an intense tourist activity takes place during the summer. In January 2015 an intense toxic cyanobacteria bloom occurred in the Uruguayan bank of the Río de La Plata river, and certain beaches were disqualified for bathing [21]. The official report drawn up by the authorities of Montevideo city indicates the sampling points studied and records the results as mean and/or maximum values, mostly without specifying which beach they correspond to [21]. For Malvín and Carrasco beaches the sampling points informed were 34°53′49.8′′ S 56°06′16.0′′ W and 34°53′31.0′′ S 56°03′16.7′′ W respectively. In the summer season 2014–2015, Carrasco and Malvín beaches were suitable for bathing and presented geometric means of fecal coliform values well below the limit of 1000 cfu·dL^−1^ established by municipal decree N 253/79 (275 cfu·dL^−1^ and 287 cfu·dL^−1^ for Malvín and Carrasco beaches, respectively). The predominant phytoplankton genus on the Uruguayan coast was *Microcystis* and, in this season, 57% of the samples studied from Montevideo beaches corresponded to the category “sampling with cyanobacteria presence but without foam”, surpassing for the first time since year 2000 the “samples without bloom” (30%). The remaining 13% corresponded to the category “sampling with cyanobacterial foam”, which were detected during the whole month of January 2015 in several beaches of Montevideo according to the official reports given weekly by the Montevideo authorities. On sampling under “sampling with cyanobacteria presence but without foam” category the mean value was 17 µg·L^−1^ for chlorophyll-a and 2.9 µg·L^−1^ for microcystins, registering maximum values of 276 µg·L^−1^ and 56 µg·L^−1^, respectively. In the case of those corresponding to the category “sampling with cyanobacterial foam” the mean and maximum values were 5600 µg·L^−1^ and 25,700 µg·L^−1^ for chlorophyll-a; and 2900 µg·L^−1^ and 8200 µg·L^−1^ for microcystins [21].

### 2.2. Patient Condition and Differential Diagnose

On admission to the Italian Hospital of Buenos Aires, the patient had grade II–III encephalopathy and required mechanical ventilator assistance. Doppler ultrasonography showed hepatomegaly and echogenic images in both lobes. Brain tomography showed cerebral edema. Laboratory features showed anemia with decreased levels of hemoglobin (8 g·dL^−1^) and hematocrit (25.4%). The patient has had a coagulopathy (INR: 3.5, PT: 26%) derived from the liver failure and serum levels of ALT (1386 IU·L^−1^), AST (1268 IU·L^−1^), total (9.4 mg·dL^−1^) and direct (4.8 mg·dL^−1^) bilirubin still increased. Albumin (2.7 g·dL^−1^) and ammonium (257 ug·dL^−1^) serum levels were also altered (Table 1).

Serological evaluation was positive for antinuclear antibodies (ANAs: 1/320) and liver kidney microsomal type 1 antibodies (LKM-1: 1/1280), and negative for anti–smooth muscle antibody, hepatitis A, B, and C viruses, Epstein-Barr virus, and cytomegalovirus.

Antinuclear antibodies (ANAs) and the liver kidney microsomal type 1 antibody (LKM-1 or CYP2D6 antibody) test is primarily used to help diagnose autoimmune hepatitis. Autoimmune hepatitis type II was diagnosed on the basis of the positive LKM-1 result. The patient was treated with three i.v. doses of 20 mg metilprednisolone·kg^−1^ and 1.5 mg cyclosporine·kg^−1^.

After a week of treatment, the patient continued with severe coagulopathy and hyperammonemia, requiring hemodialysis (Table 1). The medical staff, therefore, recommended a liver transplant and the patient was accepted on the waiting list. A liver transplant was eventually performed from a deceased donor twenty days after the affected child’s admission into the Italian Hospital of Buenos Aires.

### 2.3. Pathology Analysis of Explanted Liver

**Figure 1:**
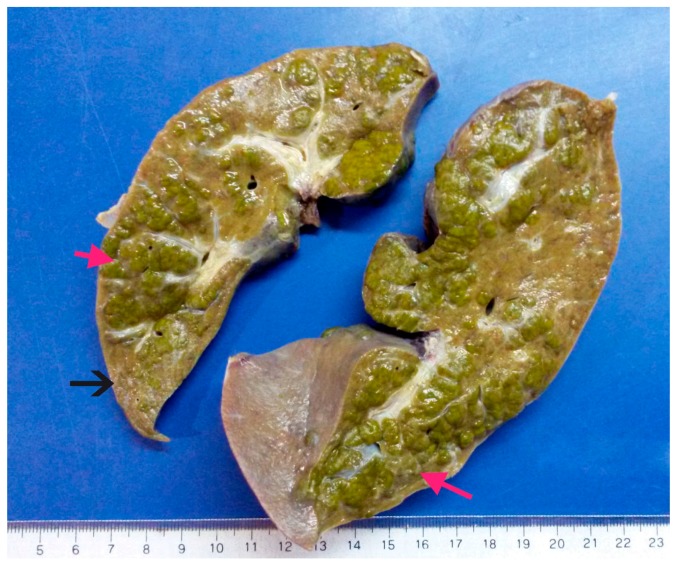
Macroscopic external appearance of explanted liver. Note the coarsely cholestatic nodular surface (red arrows) and the areas with parenchymal extinction (black arrow).

The explanted liver had a weight of 275 g and dimensions of 16 × 6 × 6 cm, including the biliary vesicle of 5 × 2 × 1.5 cm. The liver external macroscopic appearance was irregular and presented cholestasis with a coarsely nodular surface and some areas with parenchymal extinction (Figure 1).

**Figure 2:**
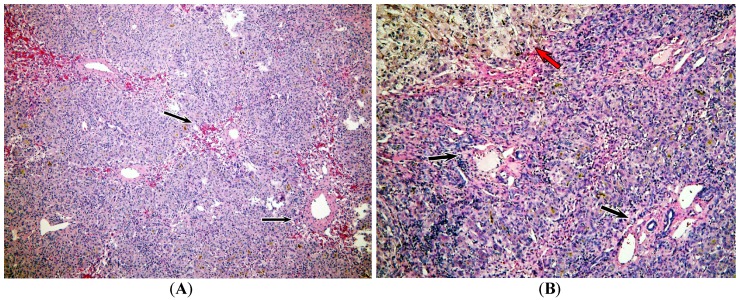
Representative slices H and E staining of the explanted liver: (**A**) Liver parenchyma with hemorrhage around central veins with hepatocyte dropout (black arrows). Vascular structures are close to each other with some degree of intracytoplasmic cholestasis (100×). (**B**) Portal tract without inflammatory activity (black arrows). Note the area of liver parenchyma with nodular regeneration and hepatocyte cholestasis (red arrow) (100×).

Representative hematoxylin and eosin (H and E)-stained slides showed a pattern of damage characterized by hemorrhage, some degree of intracytoplasmic cholestasis, zones of nodular regeneration, and a lack of inflammatory activity (Figure 2). In Figure 2A there are areas of hemorrhage around central veins and hepatocyte dropout. Large hepatocytes were also observed with ballooning and multinucleation, surrounded by a proliferation zone of small hepatocytes with an increased nucleus/cytoplasm ratio forming pseudo-acini (Figure 2B). However, bile ducts showed conserved features and no portal inflammation was evident.

**Figure 3:**
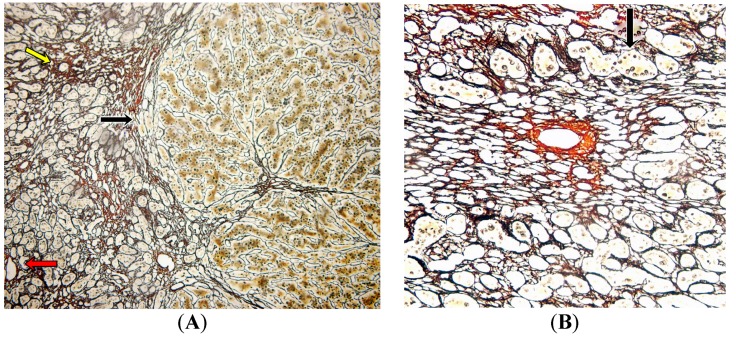
Representative slices of the reticulin-stained explanted liver. (**A**) Central vein with confluent necrosis, hepatocyte dropout (yellow arrow), and a macronodular nodule with macrotrabecular arrangement of two or three hepatocytes (black arrow). Note the portal tract without fibrosis (red arrow) (100×). (**B**) Centrilobular (zone 3) area with hepatocyte confluent necrosis and parenchymal collapsing pattern around the central vein. The black arrow shows a regenerative macrotrabecular pattern. There is no centrilobular fibrosis (400×).

Reticulin-stained sections (Figure 3) showed large areas of weft collapse around the central veins, hepatocyte dropout, and signs of regeneration with a necrosis pattern consistent with interstitial hemorrhage and parenchymal extinction. The presence of regeneration process is evidence as a macronodular nodule with macrotrabecular arrangement of two or three hepatocytes (Figure 3A). However, centrilobular fibrosis was not observed (Figure 3A,B).

Perl and Rhodamine stains, used to demonstrate iron and copper hepatocytes and cytoplasmic accumulation, respectively, were negative (Figures S1–S3).

Despite positive LKM-1 results, the liver alterations observed were not characteristic of autoimmune hepatitis type II, and at that time the cause of the patient’s condition was still not clearly established.

### 2.4. Microcystins Analysis in the Explanted Liver

**Figure 4:**
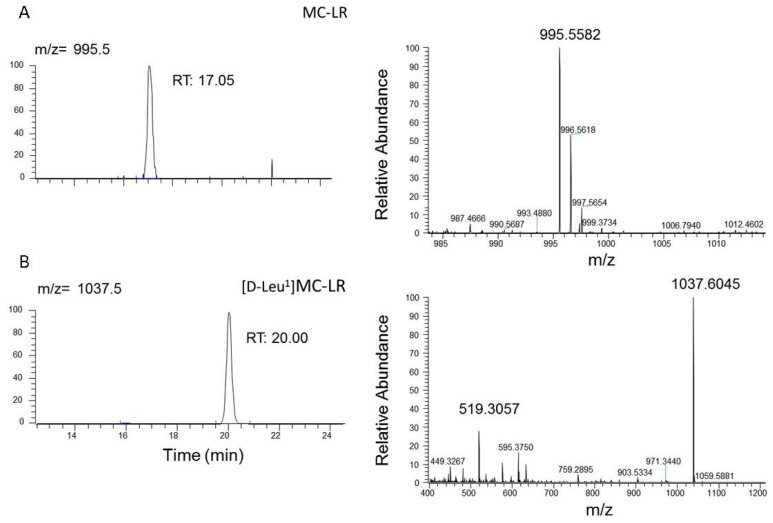
LC/ESI-HRMS analysis performed to confirm the presence of MCs in methanolic extract of the explanted liver. Selected reaction monitoring (SRM) chromatogram and product ion mass spectrum of (**A**) MC-LR with a molecular ion of 995.5582 *m*/*z* and (**B**) [D-Leu^1^]MC-LR was identified, with molecular ions of [M + H]^+^ at *m*/*z* 1037.6045 and [M + 2H]2^+^ at *m*/*z* 519.3057.

Taking into account the fact that exposure to cyanobacteria was identified as a possible cause, we carried out MCs analysis in the explanted liver using a LC-Orbitrap-MS system. A sample of 20 g of liver were extracted as we have described in the Material and Methods section and the methanolic extract was analyzed by LC/ESI-HRMS in order to evaluate the presence of microcystins in liver tissue. The analysis of chromatograms obtained from liver tissue had shown the presence of MC-LR ([M + H]^+^ *m*/*z* 995.5582) and [D-Leu^1^] MC-LR ([M + H]^+^ *m*/*z* 1037.6045) (Figure 4). The MCs nature of these molecules was confirmed by the presence of product ions of the doubly-charged [M + 2H]^+^ *m*/*z* 519.3057 and the characteristic fragments: [Arg-MeAsp-(155)-Ala-Mdha + H]^+^ *m*/*z* 595.3750 and [(M-PhCH2CH(OMe) + H]^+^ *m*/*z* 903.5334.

Additionally, we detected another fragment at *m*/*z* 135, which denotes an unmodified Adda side chain [Ph-CH_2_CH(OMe)] characteristic of microcystins. A quantitative analysis revealed the presence of 2.4 and 75.4 ng·gr^−1^ liver tissue of MC-LR and [D Leu^1^]MC-LR, respectively.

Based on these results and the pathological findings in the explanted liver, physicians performed a final diagnosis of acute liver failure related to exposure to toxic cyanobacteria and the cyanotoxin microcystin.

### 2.5. Patient’s Condition after Liver Transplant

After liver transplant the patient’s condition improved with melioration of clinical and biological parameters. After 30 days of hospitalization the infant was discharged.

At eight months post-transplant, the patient had normal clinical parameters and biological marker levels (Table 1).

## 3. Discussion

People frequently engage in recreational activities involving contact with the environment without being aware of possible adverse health effects arising from a range of contaminants present in the surroundings.

Harmful algal bloom (HAB), characterized by the exponential growth of several species of toxin-producing Cyanobacteria, frequently occurs in summer and constitutes a sanitary and environmental problem all around the world [2].

Microcystins (MCs), one of the most widely distributed groups of cyanotoxins, are part of a potent hepatotoxin group produced by genera such as *Microcystis*, *Anabaena*, *Oscillatoria*, and *Nostoc*, among others. Two of these toxins, Microsytin-LR (MC-LR) and [DLeu^1^]MC-LR, are usually present in the Río de La Plata estuary during the period of algal blooms [4,5,22].

HAB have been associated with periodic incidents of human and animal illness and death all around the world. Depending on the kind of contact that people have with cyanobacteria and their toxins, a condition characterized by different symptoms is defined. The first records of gastrointestinal diseases due to contact of the population in several cities on the Ohio River shore with cyanotoxins date from 1931 [9]. Similar alterations were observed in Harare, Zimbabwe, where children from certain areas of the city developed gastroenteritis every year coinciding with the senescence of *Microcystis* bloom [10].

One of the worst gastrointestinal toxic events, even with some lethal cases, relating to cyanotoxins, occurred in 1988 in Paulo Alfonso, Bahia, Brazil. After the construction of the Itaparica dam there was a severe epidemic of gastroenteritis associated with an *Anabaena* and *Microcystis* sp. bloom, in which about 2000 cases were reported within a period of 42 days, 88 of which were fatal; those mainly affected were children [12].

Furthermore, there are numerous reports of people who suffered symptoms, such as conjunctivitis, earache, hay fever-like syndrome, swollen lips, allergic dermatitis, rush, itching, and headache, after consuming water or coming into contact with water during a cyanobacterial bloom [23].

One of the most severe and well-documented cases of acute exposure to cyanotoxins occurred in Caruaru, Brazil, where 55 dialysis patients developed hepatic failure and died due to the use of cyanotoxin-contaminated water in the dialysis procedure. In this case, microcystins were identified in samples from the dialysis center filter, specifically in patient’s blood and post-mortem liver samples [7,8].

Another important way in which population come into contact with cyanobacterial blooms is through recreational activities. However, the effects on health of recreational MCs exposure are not yet sufficiently understood and this type of poisoning often remains undiagnosed. In previous work we reported an acute intoxication case due to recreational exposure to cyanobacteria occurring in Salto Grande Dam, Entre Ríos, Argentina in January 2007. Whilst engaging in nautical sports in this lake, a young man was immersed in an intense bloom of *Microcystis* sp. A level of 48.6 μg·L^−1^ of microcystin-LR was detected in water samples. Four hours after exposure, the patient showed nausea, abdominal pain, and fever. The initial diagnosis was stress. Three days later, dyspnea and respiratory distress were reported. The patient was hospitalized in intensive care and was diagnosed with atypical pneumonia. Finally, a week after the exposure, the patient developed hepatotoxicosis with a significant increase of hepatic damage biomarkers (ALT, AST and γGT). Complete recovery took place within 20 days [14].

In this work we report the symptoms suffered by a family after recreational activities at beaches in Montevideo, Uruguay in January 2015. Environmental monitoring of Montevideo beach water quality undertaken by the authorities of Montevideo city indicated, for January 2015, fecal coliform values below the limit of 1000 cfu·dL^−1^ and the presence of highly-toxic blooms (mainly *Microcystis*) with foam formation and average MC levels of 2.9 mg·L^−1^ and a maximum level of 8.2 mg·L^−1^ [21].

A few hours after the last exposure event, three adults developed gastrointestinal symptoms which were rapidly self-limited and a 20-month-old girl suffered gastrointestinal symptoms followed by acute liver failure, ending in liver transplant. 

Elevated levels of ALT, AST, total and direct bilirubin detected in the patient’s plasma were consistent with the observed liver damage. Likewise, these results are not surprising since the liver is the main target of microcystins and these toxins are actively transported within the hepatocytes. Thus, the inhibition of protein phosphatases, the main mechanism of action of these toxins, rapidly damages these metabolically highly-active cells.

The coagulopathy developed by the patient, characterized by alteration in PT and INR levels, is physiologically associated with the observed hepatic injury and the consequent alteration in protein synthesis factors necessary for normal functioning of the clotting and fibinolytic systems. Similar alterations in these parameters were also reported in studies which describe the biochemical outcome of Caruaru’s patients [7] and others performed on the plasma of hemodialysis patients sub-lethally exposed to microcystins [24].

The clinical parameters led to an initial diagnosis of acute liver failure, and serological evaluation had pointed to autoimmune hepatitis type II as the cause of liver failure. However, the patient did not improve after pharmacological treatment and was derived to undergo liver transplant. Liver histological studies had indicated a pattern of damage characterized by liver hemorrhagic necrosis, portal tracts without inflammatory activity and parenchyma with nodular regeneration, which is not typical for autoimmune hepatitis [25]. 

Although autoimmune hepatitis type II could have been triggered by several factors, including exposure to hepatotoxic substances, such as MCs, the damage observed at the histological level and the presence of MC-LR and [DLeu^1^]MC-LR in the liver, led us to consider a preponderant role for cyanotoxins in the development of the acute liver failure suffered by the patient.

The presence of MC-LR in the liver sample was confirmed by the retention time in comparison with MC-LR standard and the molecular ion ([M + H]^+^ *m*/*z* 995.5582) [26]. Due to the lack of [D-Leu^1^]MC-LR standard, the identification of this toxin was performed using the molecular ion ([M + H]^+^ *m*/*z* 1037.6045), the doubly-charged molecular ion [M + H]^+^ *m*/*z* 519.3057, and the main fragment ions previously reported [Arg-MeAsp-(155)-Ala-Mdha + H]^+^ *m*/*z* 595.3750 ion and [(M-PhCH2CH(OMe)) + H]^+^ *m*/*z* 903.5334 ion [26,27,28].

The toxins found in the damaged liver constitute a toxicological nexus that confirms the child’s exposure to HAB during recreational activities. We surmise that the exposure characteristics were intermittent, sub-acute for several days, and absorbed mainly via oral and dermal routes. Taking into account the MCs levels found in the child’s liver and toxin levels on Montevideo beaches (mean value of 5 mg MCs·L^−1^) reported by the authorities [21], we estimate that the girl had been exposed to the toxin contained in at least 1.78 L of the water.

Several studies were performed on mice exposed to different doses of purified MC-LR (25 µg·kg^−1^; 35 µg·kg^−1^) via i.p. Levels of toxin were detected in liver ranging from 81.1 ng·g^−1^ to 460 ng·gr^−1^ [29,30]. Even though in the current case we cannot know the exact dose and number of exposure events suffered, the values found in the liver of the patient (2.4 ng MC-LR·g^−1^ and 75.4 ng [D-Leu^1^] MC-LR·g^−1^ liver) could explain the damage observed in the liver.

Previously, micrcosystin levels in human liver were only determined in Caruaru victims. Carmichael et al. [8] reported that the average level of microcystins found in 52 liver samples from 39 fatal victims was 223 ng·g^−1^, ranging between 50.2 and 272 ng·g^−1^ estimated by ELISA assay. Consistent with this, in the present case 77.8 ng total MCs·g^−1^ of liver were found using the LC-Orbitrap-MS system.

Our findings show that exposure to toxic cyanobacteria blooms generated differential effects in adults (gastrointestinal symptoms) and a baby (acute liver failure). This difference between the pathologies presented in adults and the child may be due to: (i) the relative dose determined mainly by the duration of contact with the bloom and body size; (ii) higher body surface/body weight ratio in children than in adults, which acquires particular relevance for the dermal route; (iii) level of maturation of the liver and intestinal system and the quality of intestinal flora; and (iv) higher biological susceptibility to the development of diseases with autoimmune components.

## 4. Conclusions

In this work we have reported a case of recreational exposure to cyanobacteria and cyanotoxins, suffered by a family (three adults and a 20-month-old child) during January 2015 on the beaches of Uruguay. The adults had only self-limiting gastrointestinal symptoms while the child had more severe gastrointestinal condition resulting in acute liver failure requiring liver transplant. To the best of our knowledge this is the first report on the presence of MC-LR and [D-Leu^1^] MC-LR in liver of a patient who has undergone recreational exposure to a harmful algal bloom dominated by *Microcystis*.

This report highlights the need to encourage and promote discussion on the health assessment which focus on environmental health determinants such as toxigenic cyanobacteria bloom and their toxins. Likewise management frameworks should be generated through cooperation and co-financing in order to ensure adequate data sharing among all the sectors involved, at the same time intensifying research with a view to providing improved human health protection.

It is, therefore, necessary to include this type of hepatotoxicosis in diagnosis protocols, especially in areas affected by harmful algae blooms, so that medical staff will be aware of the pathology and be able to make a correct differential diagnosis.

Furthermore, institutional links should be established, allowing samples to be derived from hospitals and health centers to laboratories and other entities specializing in cyanotoxins in order to detect the presence of MCs in biological fluids as a confirmatory parameter.

## 5. Materials and Methods

### 5.1. Environmental Conditions of the Uruguayan Coast

Since summer 2000–2001, when toxic blooms were detected on the Montevideo coast for the first time [5], the Quality Assessment and Environmental Control Service has begun to monitor the water quality on the beaches of Montevideo in summer period on a routine basis (between 15 November and 31 March).

Routine monitoring is performed once a week at six beaches (Pajas Blancas, Cerro, Ramírez, Pocitos, Malvín, and Carrasco) independently of the presence or absence of cyanobacteria. 

Monitoring includes a visual record, by naked eye observation in real time, using three categories of visual detection: “sampling without bloom”, “sampling with cyanobacteria presence but without foam”, and “sampling with cyanobacterial foam”. Additionally, they develop bacteriological studies (fecal coliforms) and determine temperature (in situ), salinity, conductivity, turbidity, and chlorophyll-a by methods described in “Standard Methods for the Examination of Water and Wastewater” [31] and microcystins by ELISA immunoassay [32].

We employed the official data provides in the report for the summer season 2014–2015 [21] in order to summarize the water conditions of the Carrasco and Malvín beaches in the period where exposure occurred.

### 5.2. Medical Treatment and Clinical Biomarkers

The patient was attended by the medical staff of the Toxicology and Hepatology department of the Italian Hospital of Buenos Aires, Argentine, who evaluated the patient and provided the appropriate medical treatment.

Serum samples were taken and levels of alanine aminotransferase (ALT), aspartate aminotransferase (AST), total and direct bilirubin, albumin, and ammonium were determined (CMD 800 ixl, Wiener). Prothrombin time (PT) was determined (Coagulometer ACL 200, Instrumentation Laboratory, WM, Argentina) on plasma samples, obtained from blood anticoagulated with sodium citrate and the International Normalized Ratio (INR) was calculated. Additionally, serology for antinuclear antibodies (ANAs), liver kidney microsomal type 1 antibodies (LKM-1), anti-smooth muscle antibody, and for hepatitis A, B, and C viruses, Epstein-Barr virus, and cytomegalovirus were carried out in the hospital laboratory service (Architect i 1000sr, Abbott).

### 5.3. Pathology Studies

Hematoxylin and eosin (H and E)- and reticulin-stained [33,34] sections of the patient’s explanted liver were examined under light microscopy (Olympus binocular microscope) by the hospital’s pathology service. 

### 5.4. Toxicological Studies

MCs were determined in an explanted liver sample. MC extraction from the sample was carried out following the technique described by Carmichael et al. [8]. Briefly, 20 g of tissue was extracted twice with methanol, centrifuged at 7000× *g* and mixed with an equal volume of hexane. The hexane fraction was then discarded. The methanol extract was diluted with an appropriate volume of distilled water and applied to C18 silica cartridges. The 80% methanol eluate was collected [30]. The methanolic extract was analyzed in an Exactive/Orbitrap mass spectrometer equipped with an electrospray ionization (ESI) source (Thermo Fisher Scientific, Bremen, Germany). LC/ESI-HRMS (liquid chromatography electrospray ionization high-resolution mass spectrometry) analysis was performed to confirm the identity of microcystins present in the liver tissue [35]. 

## Figures and Tables

**Table 1:** Laboratory features of the patient.

Parameter	On Admission	Pre-Liver Transplant	Post-Liver Transplant	8 Months after Transplant
TB ^1^ (mg·dL^−1^)	9.4	10.8	0.3	0.3
DB ^2^ (mg·dL^−1^)	4.8	4.8	0.1	0.1
AST ^3^ (UI·L^−1^)	1268	79	31	30
ALT ^4^ (UI·L^−1^)	1386	81	60	50
INR ^5^	3.5	3.25	0.99	0.92
PT ^6^ (%)	26	14	93	100
AM ^7^ (µg·dL^−1^)	257	291	34	50

^1^ TB: Total bilirubin; ^2^ DB: direct bilirubin; ^3^ AST: aspartate aminotransferase; ^4^ ALT: alanine aminotransferase; ^5^ INR: International Normalized Ratio; ^6^ PT: prothrombin time; ^7^ AM: ammonium.

## Supplementary Materials

The following are available online at www.mdpi.com/2072-6651/9/9/267/s1, Figure S1: Representative slice H and E stained of normal liver (100X), Figure S2: Representative slices of explanted liver stained with Perls technique (100X), Figure S3: Representative slices of explanted liver stained with Rhodamine techniques (400X).
