# Supplementary material for: Recreational Exposure during Algal Bloom in Carrasco Beach, Uruguay: A Liver Failure Case Report
Source: Toxins (Basel). 2017 Aug 31;9(9):267. doi: 10.3390/toxins9090267 (PMC5618200; doi:10.3390/toxins9090267)
Supplement: Supplementary file 1 [file toxins-09-00267-s001.pdf]

# Supplementary Materials: Recreational Exposure during Algal Bloom in Carrasco Beach, Uruguay: A Liver Failure Case Report

**Flavia Vidal, Daniela Sedan, Daniel D'Agostino, María Lorena Cavalieri, Eduardo Mullen, María Macarena Parot Varela, Cintia Flores, Josep Caixach and Dario Andrinolo**

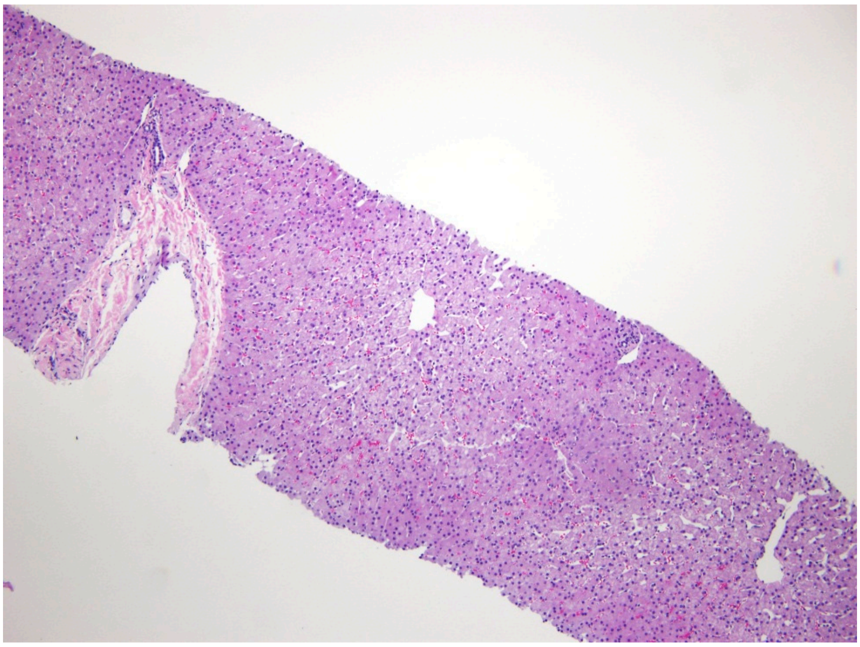

**Figure S1.** Representative slice H and E stained of normal liver (100X) shown a radial hepatocyte arrangement around the vessels characteristics of normal liver parenchyma. Note there are not hemorrhage around the central vein.

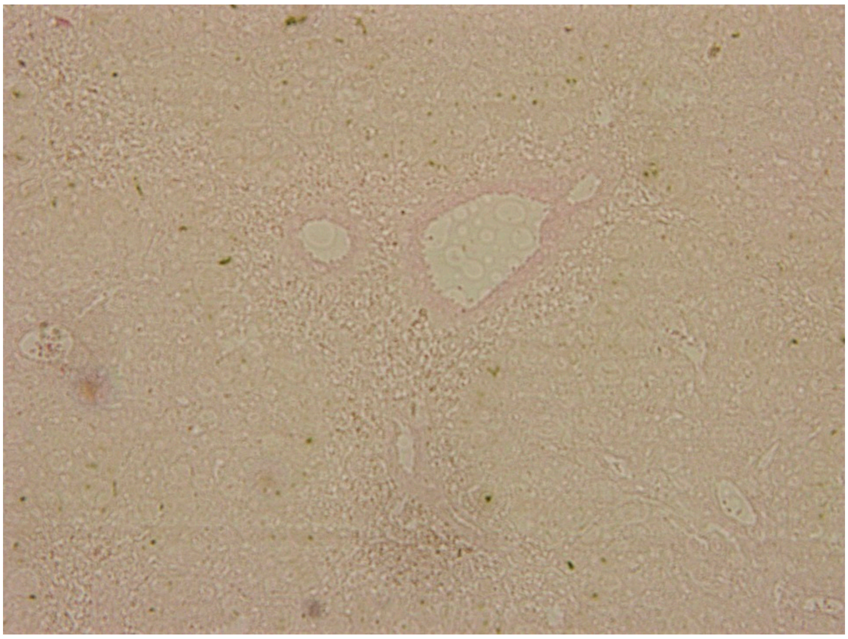

**Figure S2.** Representative slices of explanted liver stained with Perls technique (100X). Note there is no accumulation of iron in the liver parenchyma.

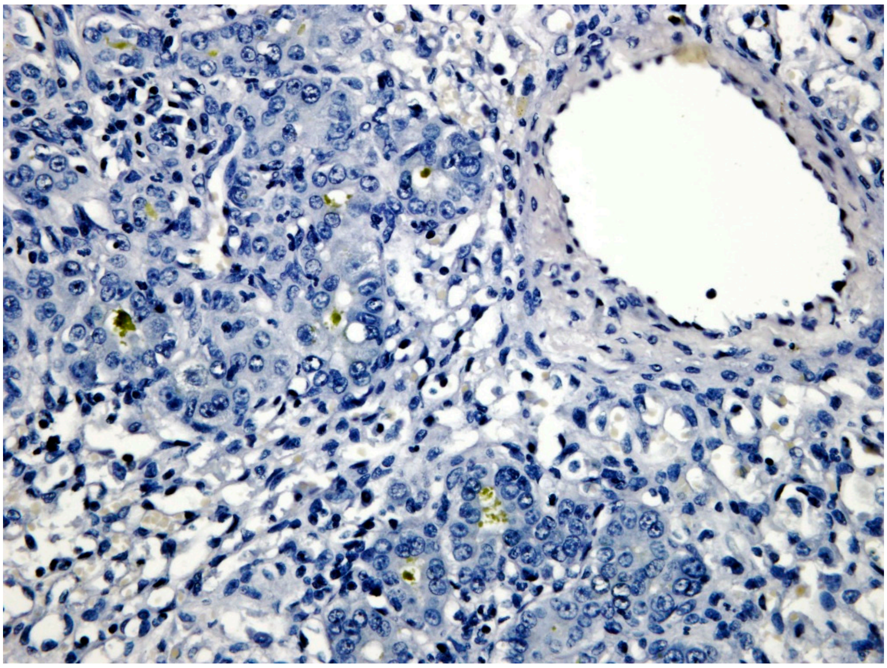

**Figure S3.** Representative slices of explanted liver stained with Rhodamine techniques (400X). The results were negative for copper accumulation in liver parenchyma.
